# Pseudoaneurysm of a Lymph Node’s Vasculature in the Axillary Tail of the Breast

**DOI:** 10.7759/cureus.88361

**Published:** 2025-07-20

**Authors:** Stephanie B Shamir, Catherine Maldjian

**Affiliations:** 1 Radiology, Montefiore Medical Center, Bronx, USA

**Keywords:** asymmetry, core needle biopsy, mammography, pseudoaneurysm of the breast, ultrasound

## Abstract

We present the rare complication of pseudoaneurysm within the vasculature of a lymph node as a result of core needle biopsy (CNB) of a lymph node within the axillary tail of the breast. The pseudoaneurysm developed within the feeding artery of a lymph node, and to our knowledge, this is the first such reported case. Pseudoaneurysm should be considered in the differential diagnosis of a mammographically new or enlarging asymmetry. Our case also emphasizes the importance of ultrasound color Doppler in establishing the correct diagnosis.

## Introduction

We present a case of iatrogenic pseudoaneurysm of an axillary tail lymph node as a result of core needle biopsy (CNB). A pseudoaneurysm is a potential iatrogenic complication of vascular access that results in compromised integrity of an arterial wall without frank rupture secondary to adjacent hematoma or soft tissues [1]. Pseudoaneurysms can further increase in size, resulting in symptoms from mass effect and ultimately rupture, which can be life-threatening [1,2]. On physical exam, a pseudoaneurysm can present as a pulsatile mass [2]. The patient in this case report presented with a mammographically enlarging asymmetry after an ultrasound-guided biopsy of a lymph node, which led to a pseudoaneurysm of the feeder vessel to the lymph node. While there are rare case reports of breast pseudoaneurysms [3-9], to our knowledge, we describe the first instance of a pseudoaneurysm in a lymph node. The diagnosis was only made possible after color Doppler ultrasound demonstrated blood flow in a saccular structure with a communicating neck between the saccular structure and the arterial vessel [1,9-11], illustrating the importance of using color Doppler imaging when interrogating “masses” on ultrasound.

## Case presentation

**Figure 1:**
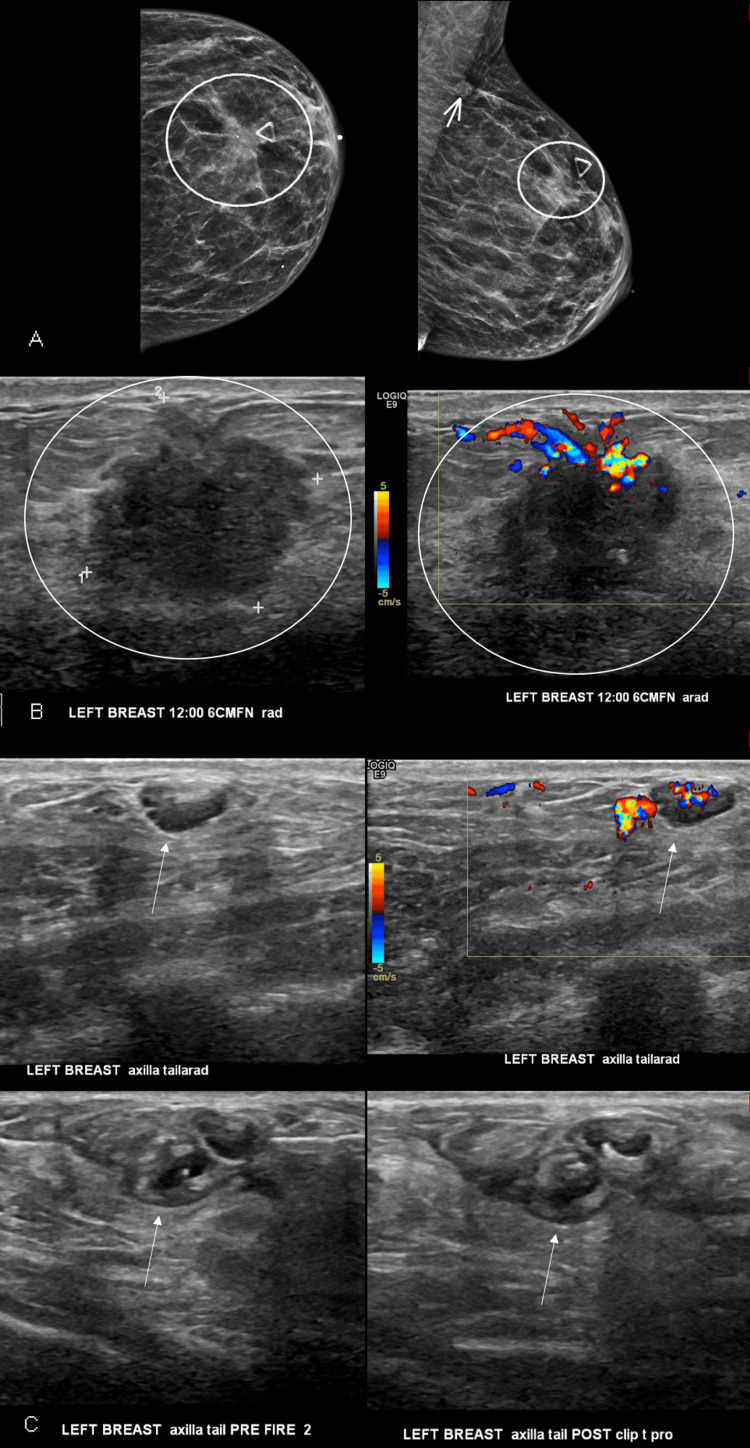
Initial diagnostic imaging and biopsy (A) Diagnostic mammogram left craniocaudal (CC) and left mediolateral oblique (MLO) images, left and right images, respectively, demonstrates an upper breast spiculated mass (circle) and axillary tail asymmetry (arrow). (B) Diagnostic ultrasound demonstrated correlates for the mammographic findings, including a spiculated mass at “left breast 12:00 6 cm FN” (top row, circles) and lymph node within the “left breast axillary tail” (bottom row, arrows). (C) Subsequent ultrasound guided biopsy demonstrates, in sequential order from left to right, bleeding of the lymph node noted immediately after the first pass and prior to the second pass, followed by imaging of the lymph node at clip placement (arrows).

A female septuagenarian patient with a history of poorly controlled hypertension and chronic kidney disease presented with a lump in the upper left breast. Diagnostic mammogram demonstrated a spiculated mass in the palpable region of concern, as well as an incidentally noted asymmetry in the axillary tail (Figure 1A). Ultrasound correlates for these findings include a 12:00 axis, 6 cm from the nipple (FN) spiculated mass, and a lymph node within the axillary tail (Figure 1B), and both findings were recommended for ultrasound-guided biopsy with clip placement. Both biopsies were performed on the same day with 14-gauge needles (exact device type is unknown). An ultrasound-guided biopsy of the mass involving four passes yielded infiltrating ductal carcinoma. An ultrasound-guided biopsy of the lymph node involving two passes was complicated by bleeding that was noted immediately after the first pass (Figure 1C). Twenty minutes of manual pressure and administration of 5 cc of lidocaine with epinephrine into the surrounding tissues for vasoconstriction were utilized. The bleeding stopped immediately after these interventions, and post biopsy mammogram was deferred. The patient remained hemodynamically stable without the need for hospitalization. The pathology of the lymph node yielded reactive changes.

**Figure 2:**
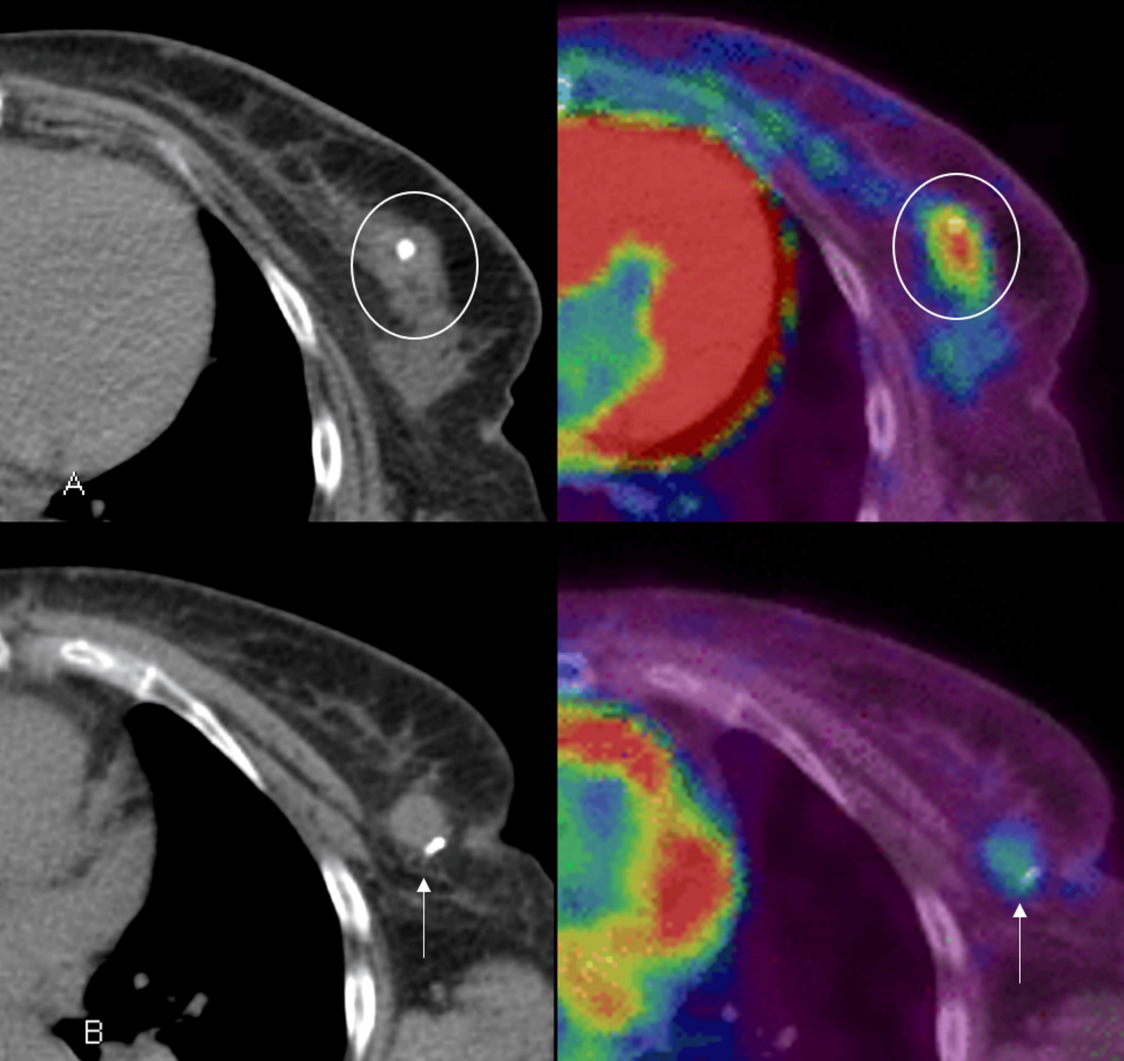
PET/CT PET/CT for the extent of the disease was performed one month after diagnosis and demonstrates the biopsy proven malignancy (A, circles), demonstrating intense radiotracer uptake (right image), and the pseudoaneurysm, seen as a soft tissue density (B, arrows), demonstrating mild radiotracer uptake (right image).

One month after diagnosis, the patient underwent a PET/CT to assess for distant metastases. In the location of the left axillary tail lymph node with a clip, a 1.6 cm soft tissue is noted with mild FDG avidity (Figure 2). The breast cancer had an SUV max of 5.7, while the left axillary tail lymph node had an SUV max of 2.8.

After the biopsy, the patient was asymptomatic. She did not mention any complaints of pain or warmth. However, four months after the original lymph node biopsy, the patient’s surgeon palpated a firm lymph node in the axillary tail. There was no mention of pulsatility.

**Figure 3:**
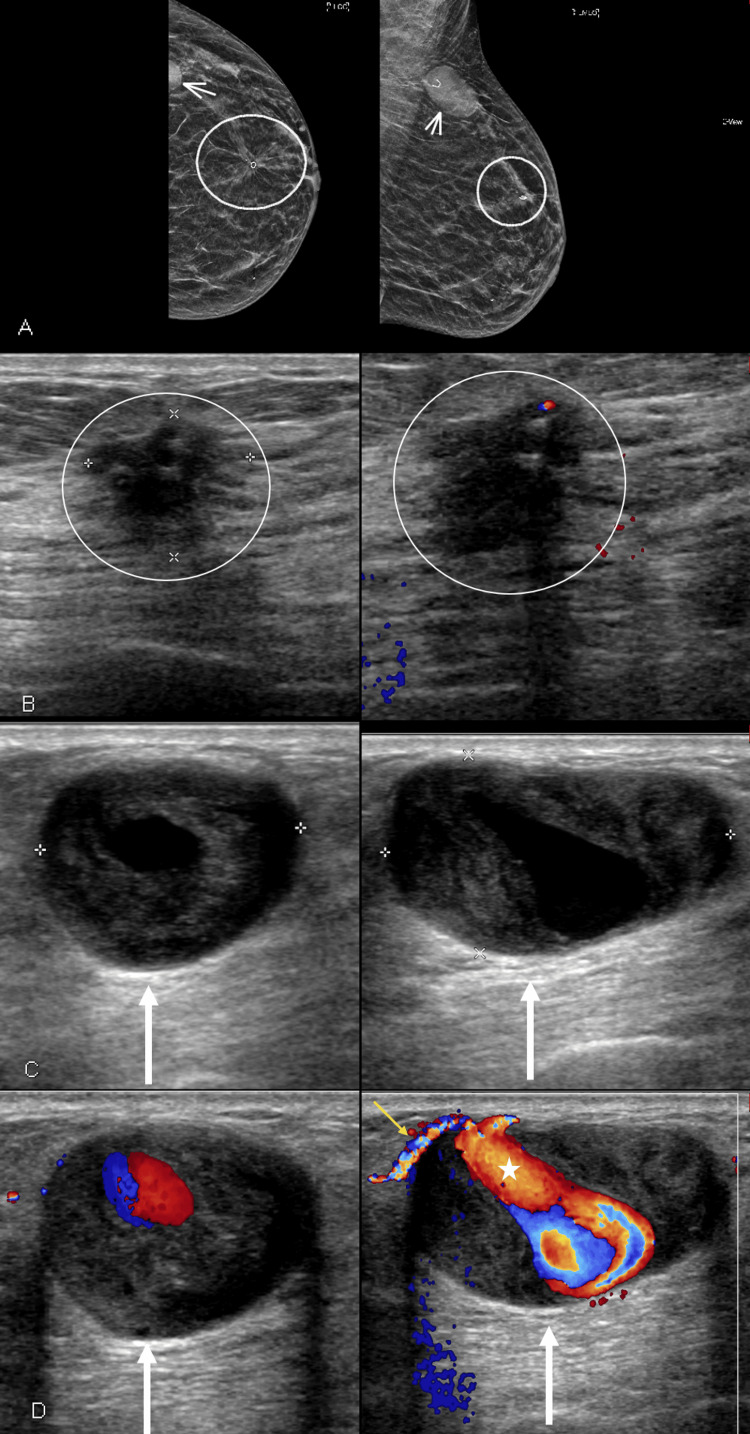
Diagnostic imaging performed five months after the ultrasound guided biopsy A. Mammogram demonstrates a significant decrease in the size of the upper breast mass (circle), but a significant interval increase in the size of the axillary tail asymmetry (arrow) compared to the prior mammogram. B. Ultrasound demonstrates the spiculated mass (circles), representing biopsy-proven infiltrating ductal carcinoma, has decreased in size compared to prior ultrasound. C. Ultrasound demonstrates a significant increase in the size of the previously biopsied lymph node (arrows). D. Doppler flow imaging of the lymph node demonstrates a “ying-yang” sign typical of pseudoaneurysm (arrows). Pseudoaneurysm neck (white star) and feeding vessel (small yellow arrow) are also noted.

Follow-up imaging performed five months later, post neoadjuvant chemotherapy, revealed that the primary cancer decreased in size, but the axillary tail lymph node now showed a cystic area of blood flow internally with a neck and with surrounding hematoma collection consistent with pseudoaneurysm (Figure 3). The axillary tail lymph node increased in size to 3.5 cm (was 1 cm prior to the biopsy), and Doppler flow imaging of the lymph node demonstrated a “ying-yang” sign typical of pseudoaneurysm. At the time of this follow-up diagnostic exam, the patient was offered treatment for the pseudoaneurysm, including graded compression and possible thrombin injection. However, ultimately, the patient and the surgeon elected for surgical removal.

The patient underwent lumpectomy for breast cancer and excision of the lymph node. The lymph node was resected utilizing palpation intraoperatively. If not for the pseudoaneurysm, the benign axillary tail lymph node would not have been resected. Pseudoaneurysm of the lymph node was confirmed on postsurgical pathology. Specifically, a benign lymph node with adjacent cystic and hemorrhagic mass and associated thick-walled vessels was seen. In totality, the lymph node with hemorrhage and organizing fibrin measured greater than 3 cm and was thought to presumptively be related to trauma and resultant disruption of a large vessel with subsequent formation of a pseudoaneurysm. The possibility of a hemangiomatous-type lesion with secondary changes related to a prior biopsy was considered a less likely diagnosis. Post surgical imaging demonstrated interval resection of the lymph node and its associated biopsy marker clip.

## Discussion

The most common complications from breast biopsies are hematomas. Pseudoaneurysms, in comparison, are extremely rare. They form as a result of transmural breach of the arterial wall with accumulation of blood in the adjacent extravascular space. This, in effect, forms a hematoma that communicates with the arterial lumen at the site of the neck or breach [3,10]. True aneurysms, on the other hand, preserve the three layers (intima, media, adventitia) of the vessel wall [4,8,9] and have been reported as a potential cause of a new breast mass [12]. A swirling pattern of flow dubbed the “yin-yang sign” is seen with pseudoaneurysm [1,9-11]. Another pseudoaneurysm infrequently encountered by breast imagers is pseudoaneurysms of adjacent vasculature in the axilla [11].

Treatment of pseudoaneurysm consists of ultrasound-guided compression, coil embolization, alcohol or thrombin injection, and surgical repair [4-6,9,10,13]. An important feature to assess successful image-guided treatment of a pseudoaneurysm is the neck length and width. Ultrasound-guided manual compression can be applied to the neck for 10-20 minutes in an attempt to arrest flow into the pseudoaneurysm in many cases [14]. In carefully selected cases, conservative management with monitoring to assess for spontaneous resolution can be attempted [8,15].

There are about 25 reports of breast pseudoaneurysms, making this an extremely rare diagnosis [3-9]. Literature search involved utilizing PubMed and multiple search terms, including “pseudoaneurysm of lymph node”, “pseudoaneurysm of breast”, and “lymph node biopsy complications pseudoaneurysm”. To our knowledge, we describe the first instance of a pseudoaneurysm within a lymph node. A similarity between our case report and some other reported incidences of iatrogenic pseudoaneurysm is the patient’s past medical history of uncontrolled hypertension [3,9,16,17]. Some instances of iatrogenic pseudoaneurysm occur in the setting of anticoagulation usage [3]; however, this patient has no history of bleeding tendencies or anticoagulation usage. In the setting of a new or enlarging “mass” after a core needle breast biopsy, the possibility of iatrogenic pseudoaneurysm should be considered, which can be diagnosed with color Doppler ultrasound.

## Conclusions

In patients with a history of previous breast biopsy, the possibility of a pseudoaneurysm should be considered in the setting of a new or enlarging “mass” or asymmetry. Our case emphasizes the importance of color Doppler in establishing the correct diagnosis. A pseudoaneurysm in a lymph node, to our knowledge, has never been previously reported.
